# Digital transformation and mental health among young adults

**DOI:** 10.3389/fpubh.2026.1800927

**Published:** 2026-05-29

**Authors:** Qin Zhang, Cuifang Du, Yuanchao Wang

**Affiliations:** 1 School of Philosophy and Social Development, Shandong University, Jinan, China; 2 School of International Cooperation and Exchange, Weifang Engineering Vocational College, Weifang, China

**Keywords:** digital transformation, internet use, mental health, social emotion, young adults

## Abstract

**Introduction:**

Digital transformation is one of the most pervasive social changes of the 21st century. Compared with children and older adults, young adults are most profoundly affected by digital transformation. This study examines the trajectory of young adults' mental health amid digital transformation, the association between internet use and young adults' mental health, and potential heterogeneity.

**Methods:**

Drawing on data from the 2013 and 2023 waves of the Chinese General Social Survey (CGSS) and official statistics from the National Bureau of Statistics of China, this study constructed a composite indicator of mental health using principal component analysis (PCA) and factor analysis (FA), and examined the association between digital transformation and young adults' mental health.

**Results:**

Against the backdrop of digital transformation, young adults' mental health declined. Internet use had a significant and robust non-linear association with young adults' mental health, with moderate use associated with better mental health. The positive association was stronger among men and urban residents, but weaker among young adults with higher educational attainment, higher socioeconomic status, and younger birth cohorts. However, once internet use became excessive, this positive association weakened substantially and began to reverse.

**Conclusion:**

In the context of digital transformation, comprehensive societal changes are associated with an overall decline in young adults' mental health. However, moderate internet use can help alleviate negative emotions and promote young adults' mental health.

## 1 Introduction

Digital transformation is one of the most pervasive social changes of the 21st century on a global scale. Compared with Europe and the United States, “super-apps” have become more fully developed in East and Southeast Asia. In China, platforms such as WeChat integrate communication, transactions, and service access within a single ecosystem (1). Moreover, the country's Digital China initiative has further embedded digital technologies into social governance and public service provision (2). For Chinese young adults, whether they welcome it or not, digital life is no longer an optional online activity but has become the very mode through which everyday life operates. Among Chinese young adults, learning, social interaction, job seeking, consumption, and self-presentation have all become increasingly intertwined with internet use.

Digital transformation does more than alter young adults' daily habits; it also reshapes how young adults relate to others and to the wider social world. As Xiang Biao's concept of the “disappearance of the nearby” highlights, under the dominance of abstract digital systems, people increasingly rely on platform-mediated information and rules, rather than on concrete people and relationships in the immediate social environment, to make sense of the world (3). Young adults occupy a life-course stage shaped by the intersection of educational tracking, job-market competition, housing pressure, decisions about marriage and childbearing. Against this backdrop, the platform economy draws constant comparison, anxiety, and emotional strain into everyday life through algorithmic recommendation, the allocation of online traffic, and competition for visibility, thereby exerting a profound influence on young adults' mental health.

In the context of digital transformation, an important question concerns how internet use associate with young adults' mental health. Existing studies, however, remain subject to several limitations. First, most prior research has focused on single, individual-level indicators, such as anxiety or depressive symptoms, loneliness, and subjective wellbeing, while paying relatively little attention to young adults' broader social attitudes and perceptions at the meso and macro levels. Second, considerable disagreement persists over the direction of the association with internet use and young adults' mental health. Some studies argue that internet use weakens family interactions and reduces the size of social networks (4), worsens sleep quality, and increases depression and loneliness among young adults (5); some even associated with cyberbullying and suicidal behaviors among young people (6). By contrast, other studies find that the negative effects of social media use on young adults' mental health are limited (7, 8), and suggest that the expansion of the internet provides young adults with more opportunities for social interaction and better fulfills psychological needs (9). Finally, existing research has yet to provide a comprehensive and systematic account of heterogeneity within the young adult population. Even when subgroup differences are considered, categorization is often limited to a single dimension, making it difficult to fully uncover the complex mechanisms through which internet use affects young adults' mental health under the intersection of multiple structural factors.

In summary, this study uses the social emotion index as a key measure of young adults' mental health and uses China as a case to examine changes in young adults' mental health in the context of digital transformation. At the individual level, digital transformation is primarily manifested in internet use. Accordingly, this study further investigates the association between internet use and young adults' mental health and identifies potential heterogeneity. Specifically, the study addresses three research questions: firstly, how has young adults' mental health changed in the context of digital transformation? Secondly, does internet use associate with young adults' mental health? Thirdly, does the association vary across different groups of young adults?

## 2 Literature review

Research on the association between digital transformation and young adults' mental health has primarily focused on specific psychological dimensions, such as self-esteem, anxiety, loneliness, and subjective wellbeing. In addition, existing studies have increasingly documented systematic differences in the association between internet use and mental health across different groups of young adults. Taken together, the existing literature can be broadly organized into the following three strands.

First, from the perspective of social support theory, internet use facilitates social interaction and exerts a positive association with young adults' mental health. Developmental theory holds that a central task of adolescence is the achievement of psychosocial autonomy (10). This process requires young people to learn self-presentation and self-disclosure (11). The anonymity, asynchronicity, and accessibility of online communication enhance adolescents' control over self-presentation and self-disclosure (12), making more young people willing to express and present themselves through the internet (13). As a form of social lubricant, social media encourage users to build weak ties across a broader range of contacts and obtain social support (14). According to a report released by the American Academy of Pediatrics, social media enable adolescents to strengthen connections with existing friends and form new friendships online, thereby reducing social isolation and loneliness and improving mental health (15). Social media use also helps maintain young adults' social capital, with especially pronounced benefits for users with low self-esteem and low life satisfaction (16).

Second, from the perspective of alienation theory, the potential risks embedded in internet use may adversely associate with young adults' mental health. Internet use may crowd out valuable time that could otherwise be spent with existing friends (17). Because online ties are often regarded as weak ties that lack emotional depth and commitment (18), users may substitute lower-quality relationships for higher-quality ones—that is, weak ties for strong ties (19). Moreover, excessive internet use and internet addiction can impair sleep quality and contribute to negative emotions such as anxiety and depression (20). Comparing one's own life with the carefully curated lives of others online can also easily trigger envy and other negative emotions (21). In addition, the internet has become a common setting and medium for cyberbullying (22). Even a single incident of cyberbullying can have serious negative consequences for young people's mental health. Research has also found a significant and consistent association between cyberbullying and depression among children and adolescents (23), pointing to its direct detrimental effect on young people's mental health (24).

Third, some scholars argue that the effect of internet use on young adults' mental health is modest, method-dependent, and heterogeneous across groups. A meta-analysis based on 67 independent samples shows that time spent on social networking sites is only weakly correlated with self-esteem, life satisfaction, loneliness, and depression (25). Other studies likewise report no significant association between adolescents' digital technology and mental health (26, 27). Using specification curve analysis and random-intercept cross-lagged panel models, studies based on eight waves of Understanding Society (the UK Household Longitudinal Study, UKHLS) data from 2009 to 2016 find that the relationship between social media use and life satisfaction is not statistically significant in more than half of the model specifications, and that this relationship varies by gender (28). Whether internet use benefits or harms young adults' mental health largely depends on the formation of meaningful social connections (29).

Overall, the literature has yet to reach a consensus on whether internet use benefits or harms young adults' mental health in the context of digital transformation, and the existing studies generally exhibits three major limitations.

First, existing studies rely on a limited set of measurement indicators for young adults' mental health. Research on the association between digital transformation and young adults' mental health has typically focused on single dimensions such as depression, anxiety, loneliness, self-esteem, and subjective wellbeing (30). These indicators are relatively fragmented and pay limited attention to holistic social mentality and emotions at the meso and macro levels.

To address this limitation, this study constructs a social emotion index as a comprehensive indicator of young adults' mental health. Social emotion refers to individuals' subjective emotional experiences that are embedded in psychological processes in social contexts and shaped through social interactions (31, 32). Emotions are not merely individual physiological or psychological responses, but are also shaped by the social environment and social networks (33). As digital transformation continues to deepen, everyday life has become increasingly intertwined with internet use, and individuals' sources of information have become more diverse and complex. This shift profoundly shapes how individuals perceive the social environment and how they assess and explain their own social standing. With the rapid circulation of information, emotions are generated and reinforced across different social groups, gradually taking the form of a collective expression that diffuses widely and resonates across society (34, 35). Group-shared social emotion can effectively reflect the state of young adults' mental health and help predict the onset of mental disorders.

**Figure 1:**
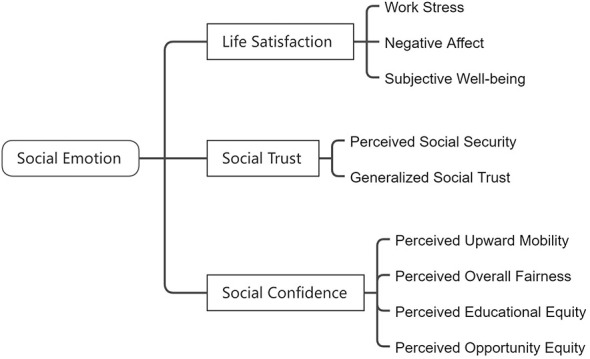
Conceptual framework of social emotion.

This study constructs a comprehensive conceptual framework of social emotion based on three core dimensions: life satisfaction, social trust, and social confidence (36). These three dimensions are further operationalized through nine specific indicators, as shown in Figure 1.

Second, the extant literature have offered diverse accounts of whether internet use benefits or harms young adults' mental health. Existing studies have examined the impact of internet use on young adults' mental health from perspectives such as information acquisition and social interaction, yet have not reached a consensus on whether internet use is associated with young adults' mental health or, if so, the direction of this association. These divergent findings mainly stem from fragmentation of research perspectives and analytical entry points.

To investigate the association of digital transformation and young adults' mental health, this study constructs three core variable—social media use intensity, leisure-time internet use intensity, and primary source of information—to characterize respondents' internet use, and assesses the association between internet use and young adults' mental health from three distinct angles: social interaction, information acquisition, and leisure-time internet use. At the same time, this study takes China as its empirical case. China has undergone the dual social transformations of institutional transition and modernization within a relatively short period of time, and therefore differs substantially from Western developed countries and transition countries in Eastern Europe in terms of trajectories of social transformation, institutional environments, and cultural traditions. Unlike the digital changes that unfolded after the completion of urbanization in Western societies, China's digitalization has taken place alongside major social transformations such as rapid urban expansion and the decline of rural communities. As a result, China's digital transformation has been exceptionally rapid, extensive, and profound. Examining the impact of internet use on young adults' mental health in the context of China's digital transformation is therefore of considerable scholarly value and practical significance.

Third, existing research lacks a comprehensive and systematic assessment of heterogeneity in the association with internet use and young adults' mental health. Most studies focus primarily on the overall association between internet use and young adults' mental health. Although some studies have noted group differences by gender, hukou status, and other characteristics, most remain centered on a single demographic factor and therefore fall short of providing a comprehensive and systematic analysis. At the same time, few studies move beyond the individual level to consider structural differences across regions and provinces among young adults, even though such social-structural variables may profoundly shape the impact of digital transformation on young adults' mental health.

To address this limitation, this study incorporates several key structural factors that are particularly informative for distinguishing subgroups of young adults, including gender, hukoustatus, educational attainment, birth cohort,^1^ and socioeconomic status (ISEI), while also incorporating provincial-level variables—Gross Domestic Product (GDP) per capita and local fiscal healthcare expenditure per capita—as macro-level factors. In this way, the study aims to explore heterogeneity in the association between internet use and young adults' mental health across multiple dimensions, including demographic characteristics, social stratification, and life-course position.

## 3 Research hypotheses

### 3.1 The trajectory of young adults' mental health in the context of digital transformation

Digital transformation entails not only the upgrading of technological tools, but also a profound reconfiguration of the way society operates and of the structure of social opportunities. According to risk society theory, modern society enhances efficiency and convenience while continuously generating new forms of uncertainty (37). From 2013 to 2023, the intensification of international political conflicts and the outbreak of the COVID-19 pandemic heightened social uncertainty. In China, the restructuring of employment patterns under digital transformation, together with narratives of class immobility, has placed growing pressure on individuals' employment prospects and everyday life. Changes in modes of social governance and systems of evaluation have made datafication, quantification, and visualization increasingly important criteria for assessing individuals. As a result, young adults are increasingly drawn into standardized and comparable evaluative frameworks in education, recruitment, performance appraisal, and social competition, intensifying the penetration of external evaluation into individual lives. Meanwhile, the wide circulation of information in the digital era has also greatly heightened public perceptions of social risk.

Accordingly, this study proposes *Hypothesis* 1: In the context of digital transformation, young adults' mental health exhibits a downward trend.

### 3.2 The association between internet use and young adults' mental health

This study argues that the association between internet use and young adults' mental health does not follow a simple linear pattern; rather, it is characterized by non-linearity. From the perspective of social support theory, moderate internet use can broaden young adults' social networks, help sustain weak-tie connections, and provide emotional support and social recognition. Through online expression and interaction, it can also strengthen young adults' sense of belonging and participation. For young adults in the stage of identity formation and social integration, the internet can, to some extent, function as a source of social support, helping to alleviate loneliness, helplessness, and pressures in everyday life, thereby exerting a positive association with young adults' mental health.

At the same time, once internet use exceeds a moderate level, its positive association may diminish and even turn negative. Excessive internet use not only crowds out time for offline interaction and rest, making social relationships more superficial, but also reinforces information cocoons and social comparison under algorithmic recommendation. Continued exposure to others' curated self-presentations, highly emotional content, and competitive narratives can readily generate feelings of relative deprivation, anxiety, and powerlessness, thereby undermining young adults' life satisfaction, social trust, and social confidence.

In other words, when used in moderation, the internet can facilitate access to resources and provide emotional support; when used excessively, however, it may intensify the pressures faced by young adults and undermine mental health. Based on this reasoning, this study proposes *Hypothesis* 2: Moderate internet use is positively associated with young adults' mental health, whereas excessive internet use is negatively associated with young adults' mental health.

## 4 Methods

### 4.1 Data

**Figure 2:**
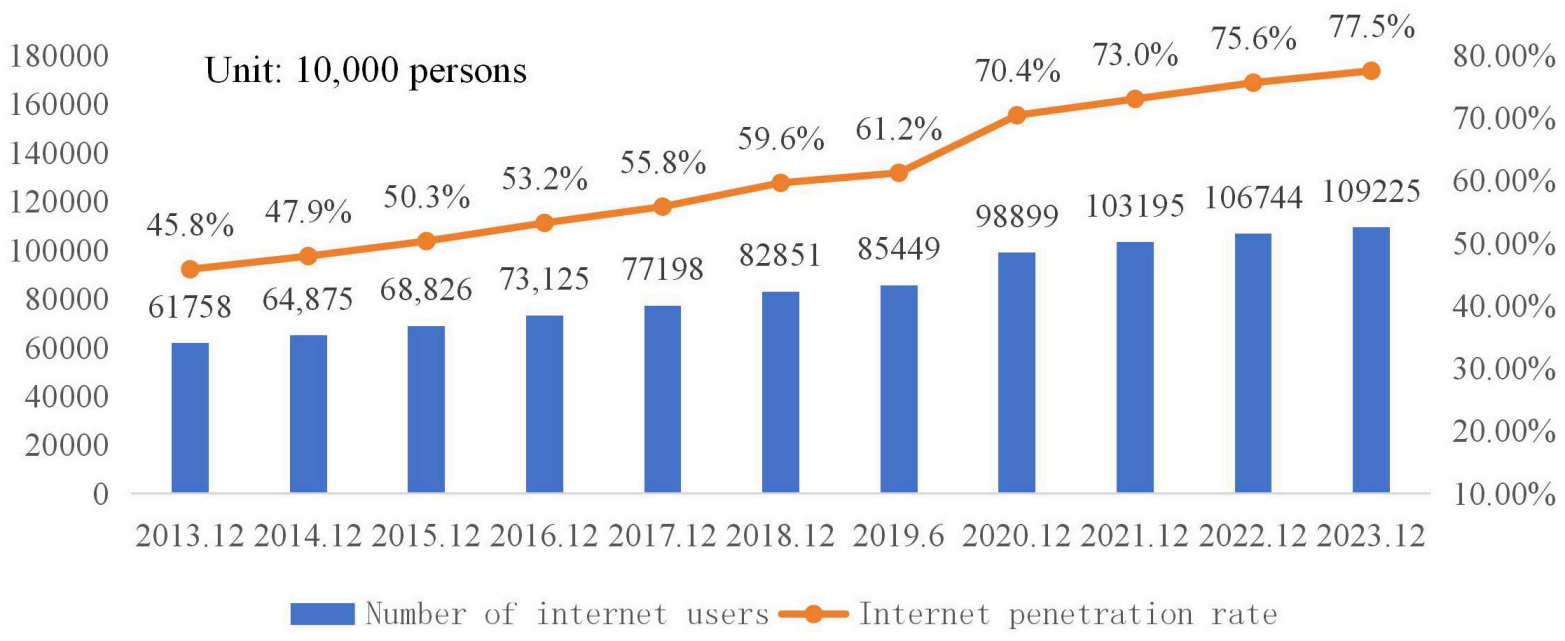
Trends in the number of internet users and internet penetration in China, 2013–2023.

This study primarily draws on large-scale nationally representative survey data from China and supplements the analysis with official statistics from the National Bureau of Statistics of China to capture relevant macro-level variables. As shown in Figure 2, both the number of internet users and the internet penetration rate in China increased substantially between 2013 and 2023.^2^ Against this backdrop, this study uses data from the 2013 and 2023 waves of the Chinese General Social Survey (CGSS). Administered by Renmin University of China, the CGSS employs a multistage stratified random sampling design and systematically collects data at multiple levels, including society, community, household, and individual. As one of China's earliest nationwide, comprehensive, and continuous academic survey programs, the CGSS provides high-quality data foundation for analyzing social emotion among Chinese young adults. This study defines young adults as individuals aged 18–40. After excluding cases with missing values and removing outliers, the final analytic samples consists of 3,897 respondents in 2013 and 4,220 respondents in 2023.

In addition to the individual-level data, this study also collects annual provincial-level macro data published by the National Bureau of Statistics of China and constructs two province-level variables: GDP per capita and local fiscal healthcare expenditure per capita. GDP per capita follows the official statistical definition, while local fiscal healthcare expenditure per capita is calculated by dividing provincial local fiscal healthcare expenditure by the total provincial population in the corresponding year. These province-level variables are matched to the CGSS individual-level data by respondents' province and survey year in order to capture regional differences in macro-level contexts. Descriptive statistics are reported in Table 1. Because different models impose different requirements regarding the completeness of key explanatory variables and control variables, the effective regression sample size may be smaller than the sample size reported in the descriptive statistics; the exact sample size is reported in each regression table.

**Table 1:** Descriptive statistics of respondents' demographic characteristics.

Variable	Category	Percentage/Mean	
		2013 (*N* = 3,897)	2023 (*N* = 4,220)
Individual-level variables			
Gender	Male	48.4	57.0
	Female	51.6	43.0
Hukou status	Urban	45.3	50.9
	Rural	54.6	48.2
	Other	0.1	0.9
Ethnicity	Han	90.5	91.0
	Ethnic minority	9.5	8.9
Educational attainment	Primary school or below	13.4	2.39
	Junior high school	30.3	13.3
	Senior high school	23.9	14.6
	College and above	32.4	69.6
	Other	0.03	0.02
Cohort	Born in the 1970s	38.6	–
	Born in the 1980s	43.6	36.8
	Born in the 1990s	17.8	44.1
	Born in the 2000s	–	19.0
ISEI	Range (20-80)	37.1	48.4
Provincial-level variables			
GDP per capita	10,000 yuan/person	4.4403	9.0931
Local fiscal healthcare expenditure per capita	Yuan/person	692.5	1,871.2

### 4.2 Variable construction

#### 4.2.1 The social emotion index as a measure of mental health

To examine young adults' mental health, this study constructs a composite social emotion index based on three core dimensions—life satisfaction, social trust, and social confidence—each measured by the indicators listed in Table 2. Specifically, the life satisfaction dimension captures young adults' mental health primarily through perceived stress, emotional experience, and subjective wellbeing at the individual level. Yet mental health is shaped not only by personal emotions, but also by how individuals perceive their social environment. The dimensions of social trust and social confidence therefore assess individuals' subjective perceptions of the broader social structure and social environment at the macro level. Social trust mainly concerns perceptions of personal safety and trust, reflecting individuals' sense of social order and social stability as well as the basic psychological need for security. Social confidence, by contrast, extends beyond security to encompass developmental psychological needs. Confidence is an important indicator of mental health (38) and reflects young adults' affirmation of both their own capabilities and the availability of avenues for social mobility.

**Table 2:** Indicators for the social emotion index.

Dimension	Observed variable	Survey item (indicator)	Coding/operationalization
Life satisfaction	Work stress	“My work is always stressful.”	1–4: strongly agree—strongly disagree
	Negative affect	“In the past 4 weeks, how often have you felt depressed or down?”	1 = always, 2 = often, 3 = sometimes, 4 = rarely, 5 = never
	Subjective wellbeing	“Overall, how happy do you feel about your life?”	1–5: very unhappy—very happy
		“I feel comfortable and at ease now, with little in my life that makes me anxious”	1–4: strongly agree—strongly disagree
		“Considering your situation, how do you think your life compares with that of an ‘average person'?”	1 = better than the average person's, 2 = about the same as the average person's, 3 = worse than the average person's
Social trust	Perceived social security	“Generally speaking, do you agree that in this society, if you are not careful, others will try to take advantage of you?”	1–5: strongly disagree—strongly agree
	Generalized social trust	“Generally speaking, do you agree that in this society, most people can be trusted?”	1–5: strongly disagree—strongly agree
		“Generally speaking, how much do you trust strangers in today's society?”	1–5: do not trust at all—trust completely
Social confidence	Perceived upward mobility	“Where do you think you will rank on the social ladder in 10 years?”	1–10: 1 (lowest)—10 (highest)
	Perceived overall fairness	“Overall, how fair do you think today's society is?”	1–5: completely unfair—completely fair
	Perceived educational equity	“As long as children work hard and are smart enough, they will have equal opportunities for further education”	1 = agree, 2 = disagree
	Perceived opportunity equity	“In our society, the children of workers and farmers have just as many opportunities as others to become wealthy and achieve high social status”	1 = agree, 2 = disagree

Response scales follow the original CGSS coding; we only applied reverse coding where necessary to ensure that higher values indicate better mental health.

In constructing the social emotion index, principal component analysis (PCA) is first applied to the observed indicators for each dimension, and the first principal component is used to derive three latent variables—life satisfaction, social trust, and social confidence.^3^ When these three latent variables are incorporated into a factor analysis (FA) model, only a single common factor has an eigenvalue greater than 1 under the Kaiser criterion, suggesting that the three dimensions share a higher-order latent structure. Shapley, decomposition is then used to equitably apportion the variance explained by the common factor across the three latent dimensions, thereby providing an objective assessment of each subdimension's contribution to social emotion and its corresponding weight. Finally, the social emotion index is constructed as the weighted sum of the three latent variables, as follows:


$$\begin{array}{r} Social\;Emotion\;=\;\alpha_{1}\;\times \;Life\;Satisfaction\;+\;\alpha_{2}\;\times \;Social\;Trust\\ +\;\alpha_{3}\;\times \;Social\;Confidence \end{array}$$

Here, α_1_ denotes the weight for the corresponding dimension, as determined by the Shapley decomposition. We further rescale the social emotion index to a range of 0–100 using min–max normalization, thereby improving interpretability and facilitating comparisons across groups and over time.

#### 4.2.2 Internet use

At the individual level, digital transformation is manifested primarily through internet use. To examine the association between digital transformation and young adults' mental health, this study constructed three core variables to capture respondents' internet-use patterns: social media use intensity, leisure-time internet use intensity, and primary source of information. In the main analysis, social media use intensity is used as the key explanatory variable. Given the possibility of a non-linear relationship between social media use and mental health, this study further includes the mean-centered linear term of this variable and its quadratic term. In the robustness checks, leisure-time internet use intensity and primary source of information are used, respectively, as alternative key explanatory variables.

Social media use intensity. This variable is based on the question, “In the past year, how often did you use social media?” Response options include “never,” “rarely,” “sometimes,” “often,” and “very often.” We treated this measure as a continuous variable ranging from 1 to 5, with higher values indicating more frequent social media use.

Leisure-time internet use intensity. This variable is derived from the question, “In the past year, how often did you engage in the following activities during your leisure time?” Response options include “every day,” “several times a week,” “several times a month,” “several times a year or less,” and “never.” We treated this measure as a continuous variable ranging from 1 to 5, with higher values indicating more frequent internet use during leisure time.

Primary source of information. This variable is based on the question, “Among the media listed above, which is your primary source of information?” Respondents who selected “the internet” are coded as 1, and all other responses are coded as 0.

### 4.3 Model

The data used in this study have a nested province–individual structure. In this type of data, lower-level units (individuals) within the same higher-level unit (province) may exhibit clustering due to within-group similarity. When within-group correlation is too high, estimation and statistical inference may be biased, making multilevel modeling necessary. To assess this issue, we estimated an unconditional null model under the multilevel nested structure and calculated the intraclass correlation coefficient (ICC). The results show that the ICC is below 0.024, indicating a low level of within-group dependence and suggesting that multilevel modeling is unnecessary. Accordingly, this study employs ordinary least squares (OLS) regression to estimate the association between digital transformation and young adults' mental health, measured by the social emotion index. Because the social emotion index is a continuous variable ranging from 0 to 100, OLS is an appropriate baseline estimation method.

Moreover, the relationship between internet use and young adults' mental health may not be strictly linear. Moderate use may be associated with positive mental health, whereas excessively frequent use may be associated with poorer mental health. To test for this possible non-linear relationship, this study includes both the mean-centered linear term of social media use intensity and its squared term in the model. Mean-centering helps reduce multicollinearity between the linear and quadratic terms and improves the clarity of interpretation. Accordingly, the model is specified as follows:


$$\begin{array}{l} Y_{i}=\beta_{0}+\beta_{1}X_{i}+\beta_{2}X_{i}^{2}+\beta^{\prime }D_{i}+\gamma^{\prime }Z_{pt}+\lambda_{t}+\varepsilon_{i} \end{array}$$

Where *Y*_*i*_ denotes the social emotion index of young adults. *X*_*i*_ denotes the mean-centered internet use intensity, and $X_{i}^{2}$ its squared term. *D*_*i*_ represents individual-level control variables, including gender, hukou type, educational attainment, birth cohort, socioeconomic status, and ethnicity. *Z*_*pt*_ denotes province-year-level macro variables, including GDP per capita and local fiscal healthcare expenditure per capita. λ_*t*_ denotes survey-year fixed effects; and ε_*i*_ is the random error term. For social media use intensity and leisure-time internet use intensity, both the mean-centered linear term and the squared term are included in the model. By contrast, primary source of information, as a binary variable, is included only in linear form.

## 5 Findings

### 5.1 Changes in young adults' mental health in the context of digital transformation

**Figure 3:**
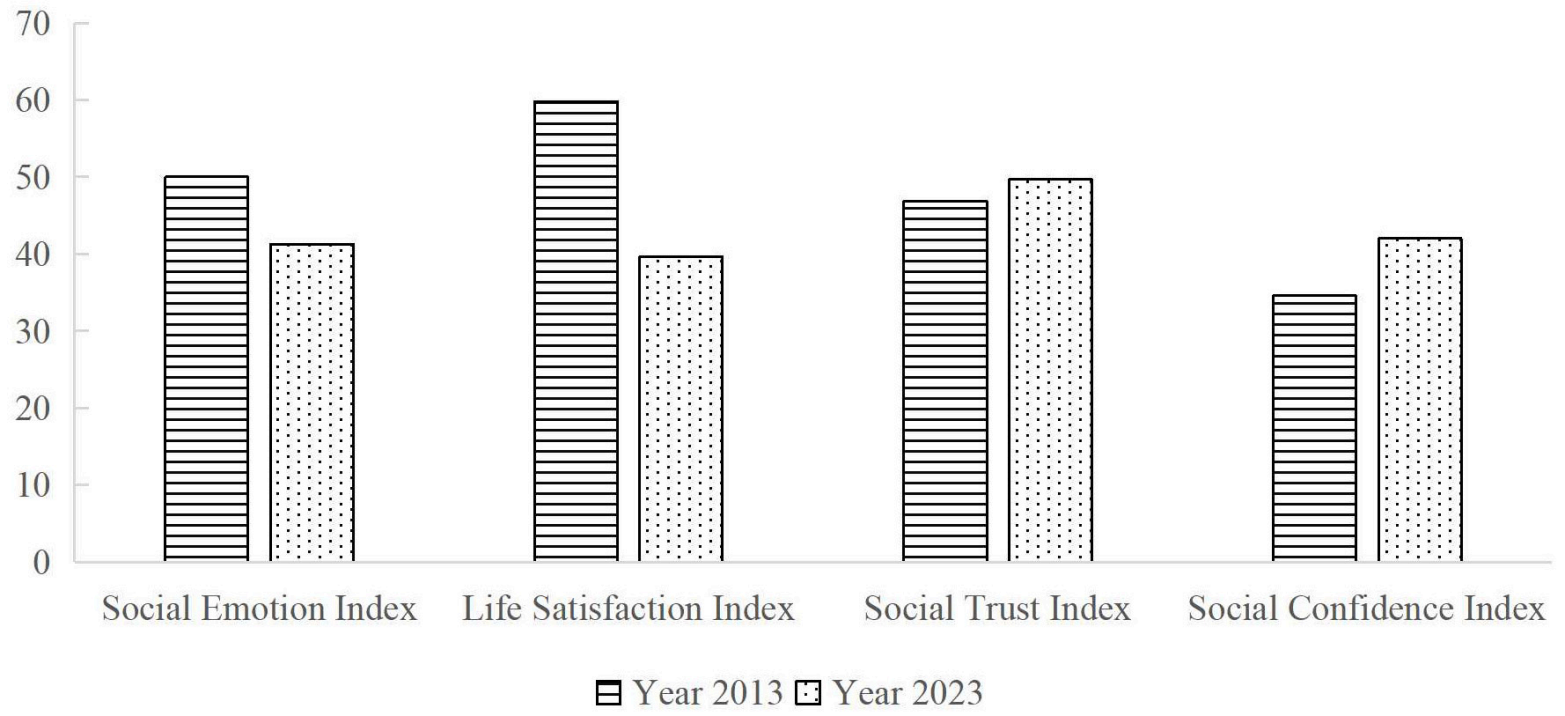
Differences in the dimensions of social emotion between 2013 and 2023.

**Figure 4:**
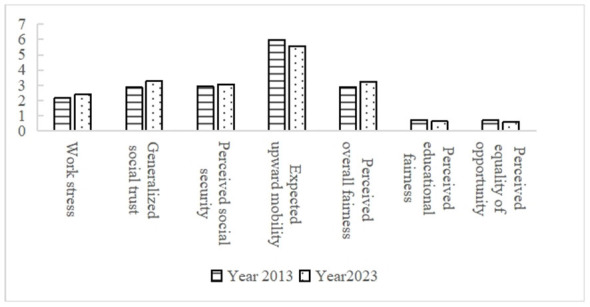
Changes in the indicators of each dimension of social emotion between 2013 and 2023.

Compared with 2013, young adults' mental health in 2023 declined significantly (*t* = 31.05, *p* < 0.001; see Figure 3), supporting Hypothesis 1. Across the three dimensions, the life satisfaction index decreased significantly in 2023, whereas the social trust and social confidence indices increased significantly (see Table 3 and Figure 4). On the one hand, declining life satisfaction reflects tensions between rising aspirations and mounting objective pressures. On the other hand, amid China's governance modernization and continued efforts to promote social equity, young adults reported more positive expectations regarding national development and their own future prospects. Changes in the specific indicators are reported in Table 4.

**Table 3:** Comparison of the social emotion index and its dimensions, 2013 vs. 2023.

Comparison item	Social emotion	Life satisfaction	Social trust	Social confidence
2013	50.0022	59.7807	46.8734	34.5726
2023	41.2679	39.6430	49.6783	42.0111
Direction of change (2023 vs. 2013)	–	–	+	+
Significance of difference	*p* < 0.001	*p* < 0.001	*p* < 0.001	*p* < 0.001

**Table 4:** Indicator-level differences in the social emotion index, 2013 and 2023.

Comparison item	Life satisfaction			Social trust			Social confidence		
	Negative affect	Subjective wellbeing	Work stress	Generalized social trust	Perceived social security	Expected upward mobility	Perceived overall fairness	Perceived educational fairness	Perceived equality of opportunity
									
2013	1.901	2.685	2.174	2.885	2.917	5.990	2.878	0.745	0.698
2023	2.445	2.844	2.403	3.293	3.075	5.568	3.229	0.641	0.598
Direction of change (2023 vs. 2013)	+	+	+	+	+	–	+	–	–
Significance of difference	*p* < 0.001	*p* < 0.001	*p* < 0.001	*p* < 0.001	*p* < 0.001	*p* < 0.001	*p* < 0.001	*p* < 0.001	*p* < 0.001

Young adults shoulder high expectations from their families. At the same time, internet use exposes them to a large volume of highly curated narratives of success, which can heighten expectations about their own lives and career trajectories. Meanwhile, China's economic reforms are entering a more complex phase. Growth in traditional industries has slowed, the stability of emerging sectors has yet to be fully consolidated, and job-market competition for young adults remains intense, with stable and well-paid positions in short supply. This mismatch between heightened expectations and mounting real-world pressures may help explain the significant decline in the life satisfaction index.

Meanwhile, young adults increasingly recognize achievements in governance modernization, basic social provision, and public safety. This pattern is consistent with the observed increases in perceived social trust and security. They also express greater optimism about pathways for upward mobility and hold more positive expectations about both national development and their own futures. This coexistence of affirmation and anxiety suggests that the gap between objective social progress and young adults' subjective sense of gain has not yet been fully bridged.

#### 5.1.1 Life satisfaction index

In 2023, even though the Young adults' life satisfaction index shows an overall declining trend, the subjective wellbeing among young adults in China increased significantly (*t* = 14.94, *p* < 0.001). This change coincided with improvements in basic living standards and the continued strengthening of the social security system accompanying China's socioeconomic development. At the same time, the broader global context has become more volatile, as complexity, instability, and uncertainty increasingly overlap. As China's economy enters a critical phase of structural adjustment, young adults face concrete pressures related to employment, income, and housing. Negative emotions among young adults were significantly higher in 2023 than in 2013 (*t* = 25.57, *p* < 0.001). The work-stress index also increased significantly over the same period (*t* = 9.13, *p* < 0.001).

Against the backdrop of rapid modernization, China's social structure has undergone rapid change, while institutional arrangements have not always kept pace. Across key life-course transitions—from intense competition for educational advancement, to performance evaluation in the early career stage, and then to the successive demands of housing, marriage and childbearing, and later-life support—young adults face rising responsibilities and expectations.

The diffusion of internet-based social media has further reshaped how information is produced and circulated, contributing to a sharp increase in the volume of social information. As information overload becomes more pronounced, young adults may experience heightened perceptions of uncertainty and greater psychological strain, which can foster more negative emotional states (39). In addition, highly visible functions on social media—such as commenting, liking, and sharing—provide immediate and transparent social feedback. These features can intensify social comparison and facilitate the spread of anxiety (40).

#### 5.1.2 Social trust index

From 2013 to 2023, both perceived social trust and perceived social safety increased significantly (see Table 4). Young adults reported greater confidence in the orderliness of social order and public safety, and offered more favorable assessments of others' trustworthiness. These shifts are consistent with broader efforts to strengthen state capacity and expand rule-of-law–based governance in China. In 2013, the Third Plenum of the 18th Central Committee of the Communist Party of China (CPC) set out the goal of modernizing the national governance system and governance capacity. Over the past decade, reforms have focused on strengthening social governance institutions and expanding rule-of-law–based governance across levels and domains, while also broadening civic participation, improving public safety, and resolving social disputes through institutional and legal channels. During this period, China's governance framework and rule-of-law system have continued to develop, providing institutional foundations that can support improvements in perceived safety and social trust.

#### 5.1.3 Social confidence index

From 2013 to 2023, young adults reported a significant increase in overall perceived fairness (*t* = 13.59, *p* < 0.001). However, perceived upward mobility declined significantly (*t* = −9.67, *p* < 0.001), as did perceived fairness in education (*t* = −8.11, *p* < 0.001) and perceived fairness in opportunities (*t* = −7.52, *p* < 0.001). On the one hand, China has continued to advance initiatives aimed at promoting social equity, supported by a set of wide-ranging and targeted policy measures. The rise in overall perceived fairness is consistent with progress in institutional efforts to enhance fairness. Although objective social stratification persists, young adults appear to evaluate the overall fairness of the system relatively positively. On the other hand, the declines in perceived upward mobility, educational fairness, and opportunity fairness suggest that improvements in overall fairness perceptions have not translated into confidence in one's own prospects for upward mobility. This pattern may be shaped by intensified competition, heightened occupational uncertainty, and mounting pressures associated with the cost of living.

### 5.2 The association between internet use and young adults' mental health

Tables 5–8 report the regression results for the social emotion index and the indicators for its component dimensions. In models 1–5, this study sequentially introduced controls for survey year, gender, birth cohort, hukou status, educational attainment, socioeconomic status, and province-level macro variables. The results show a significant and robust non-linear relationship between internet use and young adults' social emotion index, following an overall inverted U-shaped pattern. This finding supports Hypothesis 2: moderate internet use is positively associated with young adults' mental health, whereas excessive internet use is negatively associated with young adults' mental health. In addition, young adults who rely on the internet as their primary source of information have significantly higher social emotion index scores than those who rely primarily on other channels.

**Table 5:** Relationship between social media use intensity and the social emotion index among young adults.

Variables	Social emotion index				
	Model 1	Model 2	Model 3	Model 4	Model 5
Linear term of social media use intensity	0.545^***^	0.468^**^	0.380^*^	0.105	0.036
	(0.202)	(0.208)	(0.208)	(0.214)	(0.216)
Quadratic term of social media use intensity	−0.415^***^	−0.408^***^	−0.386^***^	−0.350^***^	−0.351^***^
	(0.104)	(0.104)	(0.104)	(0.106)	(0.106)
Year 2023	−7.910^***^	−7.515^***^	−7.796^***^	−8.316^***^	−8.377^***^
	(0.365)	(0.430)	(0.435)	(0.455)	(0.712)
Male		0.176	0.176	0.211	0.212
		(0.327)	(0.326)	(0.326)	(0.326)
Birth cohort (ref: 1970s)					
1980s		−1.008^**^	−0.966^**^	−1.080^**^	−1.028^**^
		(0.433)	(0.432)	(0.431)	(0.431)
1990s		−0.827	−0.816	−1.119^**^	−1.018^*^
		(0.562)	(0.561)	(0.561)	(0.562)
2000s		−1.012	−1.024	−1.493	−1.356
		(1.015)	(1.014)	(1.014)	(1.014)
Urban hukou		1.332^***^	0.962^***^	0.288	0.190
		(0.346)	(0.357)	(0.381)	(0.385)
Han ethnicity		1.123^**^	1.027^*^	0.813	0.407
		(0.546)	(0.546)	(0.546)	(0.562)
Socioeconomic status index (ISEI)			0.035^***^		0.014
			(0.009)		(0.010)
Educational attainment (ref: primary school or below)					
Junior high school				1.792^***^	1.670^***^
				(0.630)	(0.631)
Senior high school				2.996^***^	2.686^***^
				(0.720)	(0.728)
Associate degree or above				4.533^***^	3.887^***^
				(0.728)	(0.774)
Log of GDP per capita					1.632^***^
					(0.519)
Log of local fiscal healthcare expenditure per capita					−1.177
					(0.729)
Constant	51.029^***^	49.911^***^	48.705^***^	47.701^***^	52.981^***^
	(0.279)	(0.702)	(0.760)	(0.873)	(4.435)
*N*	5,228	5,228	5,228	5,228	5,228

^***^, ^**^, and ^*^ denote 1%, 5%, and 10% significance levels, respectively.

Across the three dimensions of the social emotion index, internet use exhibits a significant and robust non-linear relationship with both the life satisfaction index and the social confidence index, following an overall inverted U-shaped pattern. By contrast, no robust and statistically significant non-linear relationship is found between internet use and young adults' social trust.

#### 5.2.1 Internet use and the overall social emotion index

Table 5 reports the regression results for the relationship between social media use intensity and the social emotion index among young adults. Overall, social media use intensity exhibits a relatively stable non-linear relationship with young adults' social emotion. Moderate or moderately high levels of social media use are associated with higher social emotion index scores; however, as use intensity increases further, the positive association weakens and begins to decline. In Models 1 through 3, the mean-centered linear term of social media use intensity is significantly positive, while the quadratic term is significantly negative. After further controlling for educational attainment and province-level macro variables, the statistical significance of the linear term gradually weakens and becomes insignificant in Models 4 and 5, whereas the quadratic term remains significantly negative throughout. A joint test further shows that the linear and quadratic terms of social media use intensity are jointly significant (*F* = 10.63, *p* < 0.001), indicating that this set of variables has overall explanatory power for social emotion.

**Figure 5:**
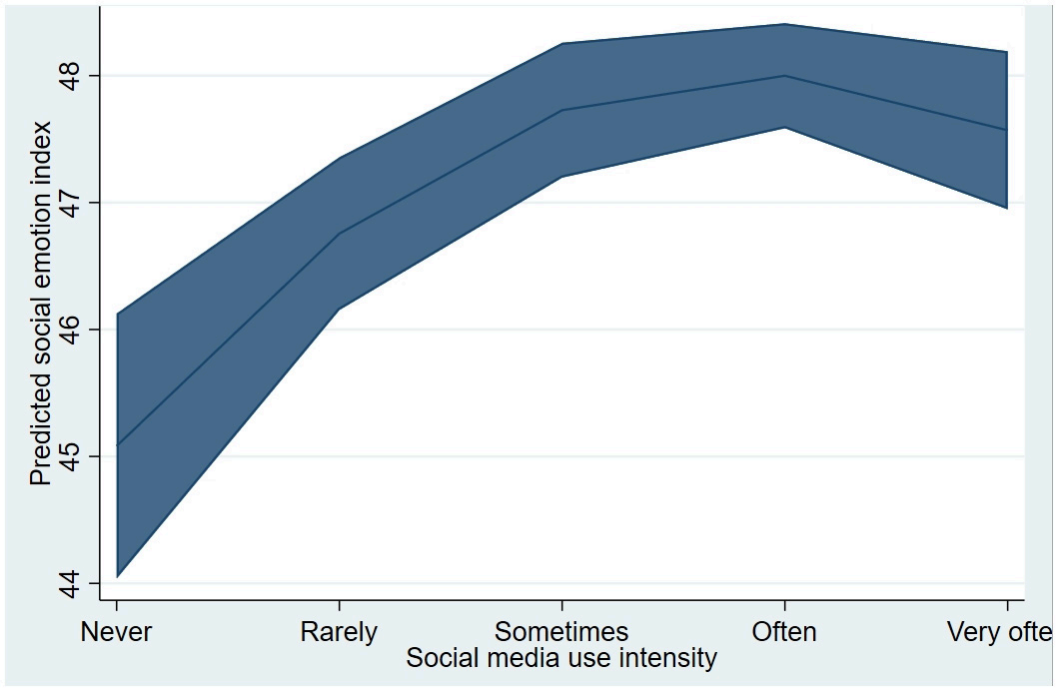
Predicted marginal relationship between social media use intensity and the social emotion index.

Figure 5 provides a clearer illustration of the relationship between social media use intensity and young adults' social emotion. As social media use intensity rises from “never” to “often,” the predicted value of the social emotion index increases steadily. However, when use frequency further reaches “very frequently,” the predicted value declines, revealing an overall inverted U-shaped pattern. According to the estimated turning point, the peak occurs at a level close to “often.” This suggests that moderate or moderately high levels of social media use may be associated with higher levels of social emotion, whereas very frequent social media use is unlikely to generate comparable emotional benefits and may even be accompanied by a decline in social emotion.

Turning to the control variables, the year effect is consistently negative and statistically significant across all models. Compared with 2013, young adults in the 2023 sample have significantly lower social emotion index scores. Among the demographic variables, gender is not statistically significant in any model, suggesting that no stable difference exists in the social emotion index between male and female young adults. In terms of birth cohort, with those born in the 1970s as the reference group, respondents born in the 1980s exhibit significantly lower levels of social emotion in all models. The coefficients for those born in the 1990s are also generally negative and become statistically significant in the later models. By contrast, the difference between those born in the 2000s and those born in the 1970s is not statistically significant. Ethnicity and hukou show some explanatory power in the baseline models: young adults with urban hukou and Han ethnicity have higher levels of social emotion than those with rural hukou and those from ethnic minority groups. However, these differences are no longer statistically significant after educational attainment and province-level macro variables are included.

The results for socioeconomic status and educational attainment suggest that these two factors do not affect young adults' social emotion in the same way. After the socioeconomic status index is introduced in Model 3 on the basis of Model 2, the coefficient for socioeconomic status is significantly positive (β = 0.035, *p* < 0.01), indicating that young adults with higher socioeconomic status tend to have higher social emotion index scores. However, in Model 5, the coefficient for socioeconomic status declines to 0.014 and is no longer statistically significant. By contrast, educational attainment exhibits a significant and stable positive gradient association in both Models 4 and 5. Using the group with primary school education or below as the reference category, young adults with junior high school, senior high school, and junior college or above all have significantly higher social emotion index scores. These findings indicate that higher educational attainment is associated with higher levels of social emotion among young adults, and that this relationship remains robust after controlling for socioeconomic status and province-level macro variables.

With respect to the province-level macro variables, Model 5 shows that the coefficient on the log of GDP per capita is significantly positive (β = 1.632, *p* < 0.01), indicating that young adults in more economically developed regions tend to exhibit higher levels of social emotion. By contrast, the coefficient on the log of local fiscal healthcare expenditure per capita is negative, but does not reach statistical significance.

The results indicate that social media use intensity has a significant and robust inverted U-shaped relationship with the social emotion index. Further robustness checks show that this non-linear relationship remains when the key explanatory variable is replaced by leisure-time internet use intensity. Based on the estimated turning point, the turning point for leisure-time internet use intensity is approximately 3.81 on the original 1–5 scale, with the peak occurring at a frequency close to “several times a week.” When the key explanatory variable is replaced by primary source of information, young adults who rely on the internet as their primary source of information have significantly higher social emotion index scores than those who rely primarily on other channels.

#### 5.2.2 Internet use and the life satisfaction index

**Figure 6:**
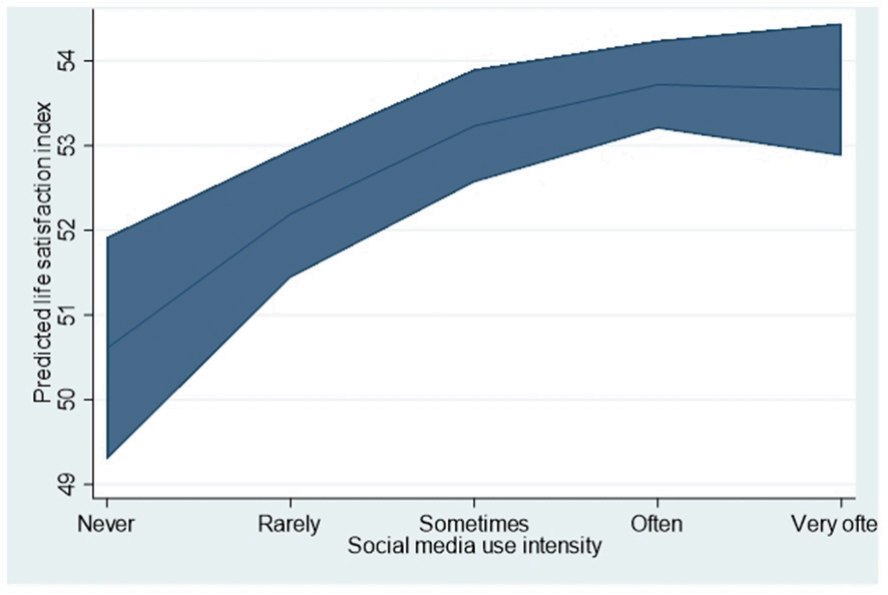
Predicted marginal relationship between social media use intensity and the life satisfaction index.

Table 6 reports the regression results for the relationship between social media use intensity and the life satisfaction index among young adults. Overall, social media use intensity exhibits a stable non-linear relationship with young adults' life satisfaction. In Models 1 through 3, the linear term of social media use intensity is significantly positive, while the quadratic term is significantly negative. After further controlling for educational attainment and province-level macro variables, the linear term is no longer statistically significant in Models 4 and 5, whereas the quadratic term remains significantly negative. A joint test further shows that the linear and quadratic terms of social media use intensity are jointly significant (*F* = 7.81, *p* < 0.001), indicating that this set of variables cannot be jointly omitted from the model. Moderate or moderately high levels of social media use are associated with higher levels of life satisfaction; however, as use intensity increases further, the positive association weakens and begins to decline (see Figure 6).

**Table 6:** Relationship between social media use intensity and the life satisfaction index among young adults.

Variables	Life satisfaction index				
	Model 1	Model 2	Model 3	Model 4	Model 5
Linear term of social media use intensity	0.553^**^	0.567^**^	0.522^**^	0.372	0.305
	(0.253)	(0.261)	(0.262)	(0.269)	(0.271)
Quadratic term of social media use intensity	−0.373^***^	−0.381^***^	−0.370^***^	−0.272^**^	−0.275^**^
	(0.130)	(0.131)	(0.131)	(0.133)	(0.133)
Year 2023	−19.615^***^	−19.287^***^	−19.428^***^	−19.486^***^	−19.196^***^
	(0.457)	(0.540)	(0.546)	(0.573)	(0.896)
Male		−0.532	−0.532	−0.562	−0.553
		(0.410)	(0.410)	(0.410)	(0.410)
Birth cohort (ref: 1970s)					
1980s		−0.136	−0.115	−0.239	−0.191
		(0.543)	(0.543)	(0.543)	(0.543)
1990s		−0.454	−0.448	−0.678	−0.580
		(0.705)	(0.705)	(0.706)	(0.707)
2000s		−0.992	−0.998	−1.300	−1.193
		(1.274)	(1.274)	(1.275)	(1.276)
Urban hukou		0.110	−0.076	−0.353	−0.418
		(0.434)	(0.448)	(0.480)	(0.484)
Han ethnicity		0.204	0.155	−0.058	−0.531
		(0.685)	(0.686)	(0.687)	(0.707)
Socioeconomic status index (ISEI)			0.018		0.009
			(0.011)		(0.012)
Educational attainment (ref: primary school or below)					
Junior high school				3.453^***^	3.320^***^
				(0.792)	(0.794)
Senior high school				3.571^***^	3.283^***^
				(0.906)	(0.916)
Associate degree or above				4.065^***^	3.538^***^
				(0.916)	(0.974)
Log of GDP per capita					1.844^***^
					(0.653)
Log of local fiscal healthcare expenditure per capita					−1.692^*^
					(0.918)
Constant	60.842^***^	61.014^***^	60.407^***^	57.988^***^	66.488^***^
	(0.349)	(0.881)	(0.955)	(1.099)	(5.581)
*N*	5,228	5,228	5,228	5,228	5,228

^***^, ^**^, and ^*^ denote 1%, 5%, and 10% significance levels, respectively.

Turning to the control variables, the survey-year effect is consistently negative and statistically significant across all models. Compared with 2013, young adults in the 2023 sample have significantly lower life satisfaction index scores, indicating that the overall level of life satisfaction among young adults declined over the 10-year period. Among the demographic variables, gender, birth cohort, hukou type, and ethnicity do not reach statistical significance in any model, suggesting that no stable differences in the life satisfaction index exist across young adults by gender, cohort, hukou type, or ethnicity.

With respect to socioeconomic status and educational attainment, Model 3 introduces the ISEI on the basis of Model 2. The coefficient on ISEI is positive but not statistically significant (β = 0.018); in Model 5, it declines further to 0.009 and remains insignificant. By contrast, educational attainment exhibits a significant and stable positive association in both Models 4 and 5. Using the group with primary school education or below as the reference category, young adults with junior high school, senior high school, and junior college or above all show significantly higher life satisfaction index scores in Model 4, revealing a clear positive gradient by educational attainment. In Model 5, the corresponding coefficients are 3.320, 3.283, and 3.538, respectively, with a slight decline for the senior high school group relative to the junior high school group. Overall, these findings suggest that higher educational attainment is associated with higher levels of life satisfaction among young adults, and that this relationship remains robust after controlling for ISEI and province-level macro variables. A further comparison of Models 3 and 4 shows that once educational attainment is included, the linear term of social media use intensity declines sharply and becomes statistically insignificant, whereas it remains significant when only ISEI is added. This suggests that educational attainment plays a more critical role than ISEI in explaining the relationship between social media use and life satisfaction.

With respect to the province-level macro variables, Model 5 shows that the coefficient on the log of GDP per capita is significantly positive (β = 1.844, *p* < 0.01), indicating that young adults in more economically developed regions tend to exhibit higher levels of life satisfaction. By contrast, the coefficient on the log of local fiscal healthcare expenditure per capita is negative and statistically significant at the 10% level (β = −1.692, *p* < 0.10), suggesting a modest negative association between local fiscal healthcare expenditure per capita and young adults' life satisfaction. The direction and underlying mechanism of this relationship require further interpretation in light of regional differences in the structure of public expenditure and the broader social environment.

The results indicate that social media use intensity has a significant and robust inverted U-shaped relationship with the life satisfaction index among young adults. Further robustness checks show that this non-linear relationship persists when the key explanatory variable is replaced by leisure-time internet use intensity. Based on the estimated turning point, the turning point for leisure-time internet use intensity is approximately 4.00 on the original 1–5 scale, corresponding roughly to a frequency of “several times a week.” When the key explanatory variable is replaced by primary source of information, young adults who rely on the internet as their primary source of information have significantly higher life satisfaction index scores than those who rely primarily on other channels.

#### 5.2.3 Internet use and the social trust index

Table 7 reports the regression results for the relationship between social media use intensity and the social trust index among young adults. With respect to the dimension of social trust, no robust and statistically significant non-linear relationship is found between social media use intensity and the social trust index. Overall, social media use intensity has relatively limited explanatory power for young adults' social trust.

**Table 7:** Relationship between social media use intensity and the social trust index among young adults.

Variables	Social trust index				
	Model 1	Model 2	Model 3	Model 4	Model 5
Linear term of social media use intensity	0.408	0.445	0.259	−0.165	−0.215
	(0.293)	(0.301)	(0.302)	(0.310)	(0.312)
Quadratic term of social media use intensity	−0.089	−0.109	−0.061	−0.096	−0.081
	(0.150)	(0.151)	(0.151)	(0.153)	(0.154)
Year 2023	2.920^***^	3.495^***^	2.902^***^	1.825^***^	1.526
	(0.529)	(0.624)	(0.630)	(0.660)	(1.033)
Male		−0.015	−0.016	0.108	0.090
		(0.475)	(0.473)	(0.473)	(0.473)
Birth cohort (ref: 1970s)					
1980s		−1.556^**^	−1.468^**^	−1.630^***^	−1.551^**^
		(0.628)	(0.626)	(0.625)	(0.625)
1990s		−1.762^**^	−1.740^**^	−2.201^***^	−2.070^**^
		(0.816)	(0.813)	(0.814)	(0.814)
2000s		−0.541	−0.567	−1.310	−1.114
		(1.474)	(1.469)	(1.469)	(1.471)
Urban hukou		0.743	−0.036	−1.148^**^	−1.298^**^
		(0.502)	(0.517)	(0.553)	(0.558)
Han ethnicity		0.457	0.254	0.019	−0.263
		(0.793)	(0.791)	(0.791)	(0.814)
Socioeconomic status index (ISEI)			0.075^***^		0.038^***^
			(0.013)		(0.014)
Educational attainment (ref: primary school or below)					
Junior high school				0.678	0.526
				(0.913)	(0.914)
Senior high school				2.709^***^	2.211^**^
				(1.043)	(1.056)
Associate degree or above				6.149^***^	4.947^***^
				(1.056)	(1.122)
Log of GDP per capita					1.091
					(0.752)
Log of local fiscal healthcare expenditure per capita					−0.538
					(1.057)
Constant	47.065^***^	47.350^***^	44.806^***^	45.505^***^	46.665^***^
	(0.404)	(1.019)	(1.101)	(1.266)	(6.430)
*N*	5,228	5,228	5,228	5,228	5,228

^***^, and ^**^ denote 1% and 5% significance levels, respectively.

Turning to the control variables, the survey-year coefficient is significantly positive in Models 1 through 4, indicating that, compared with 2013, young adults in 2023 have higher social trust index scores. However, once educational attainment, ISEI, and province-level macro variables are simultaneously included in Model 5, this difference is no longer statistically significant. The gender variable remains insignificant across all models. In terms of birth cohort, with those born in the 1970s as the reference group, respondents born in the 1980s and 1990s have significantly lower social trust index scores in Models 2 through 5, whereas the difference between those born in the 2000s and those born in the 1970s is never statistically significant. This suggests that cohort differences in social trust are mainly reflected in the relatively lower levels of social trust among those born in the 1980s and 1990s. The hukou variable is significantly negative in Models 4 and 5, indicating that, after controlling for educational attainment and other variables, young adults with urban hukou exhibit lower levels of social trust. The ethnicity variable does not reach statistical significance in any model, suggesting that no robust difference in social trust exists between Han and ethnic minority young adults.

With respect to socioeconomic status and educational attainment, both appear to have greater explanatory power for the social trust index than social media use intensity. After ISEI is added in Model 3 on the basis of Model 2, its coefficient is significantly positive (β = 0.075, *p* < 0.01). Although the coefficient declines somewhat in Model 5, it remains significantly positive (β = 0.038, *p* < 0.01), indicating that higher socioeconomic status is associated with higher levels of social trust among young adults. In terms of educational attainment, using young adults with primary school education or below as the reference group, those with senior high school education and junior college or above have significantly higher social trust index scores. This suggests that higher educational attainment is associated with higher levels of social trust, and that this relationship remains relatively robust after controlling for socioeconomic status and province-level macro variables. By contrast, neither the linear term nor the quadratic term of social media use intensity is statistically significant throughout the models. To some extent, this suggests that social trust is more closely related to factors such as educational resources and socioeconomic conditions than to the direct influence of social media use intensity.

With respect to the province-level macro variables, Model 5 shows that neither the coefficient on the log of GDP per capita nor the coefficient on the log of local fiscal healthcare expenditure per capita reaches statistical significance.

Overall, no robust and statistically significant relationship is found between social media use intensity and the social trust index among young adults. This conclusion remains unchanged when social media use intensity is replaced by leisure-time internet use intensity and primary source of information. These findings suggest that the association between internet use and young adults' mental health is not uniform across different dimensions of social emotion: internet use has a more pronounced association with the overall social emotion index and the life satisfaction dimension, but a relatively weaker association with social trust, which is a more cognitively and structurally grounded indicator.

#### 5.2.4 Internet use and the social confidence index

**Figure 7:**
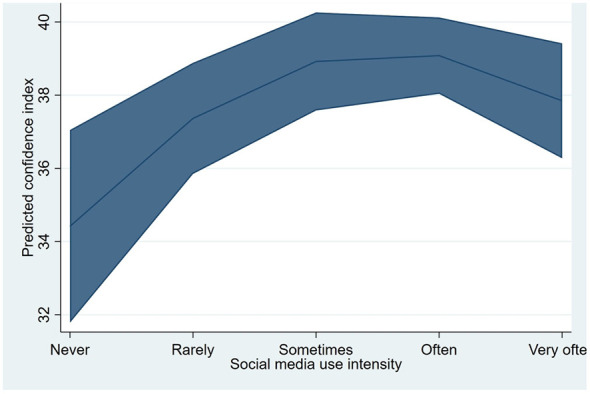
Predicted marginal relationship between social media use intensity and the social confidence index.

Table 8 reports the regression results for the relationship between social media use intensity and the social confidence index among young adults. Overall, social media use intensity exhibits a significant and relatively robust non-linear relationship with the social confidence index, following an inverted U-shaped pattern. Across Models 1 through 5, although the linear term of social media use intensity does not reach statistical significance, the quadratic term remains significantly negative throughout. A joint test further shows that the linear and quadratic terms are jointly significant (*F* = 4.67, *p* < 0.01), indicating that this set of variables cannot be jointly omitted from the model. Based on the estimated turning point, the turning point for social media use intensity is approximately 3.38 on the original 1–5 scale, falling between “sometimes” and “often,” which suggests that the peak of social confidence occurs at a moderately high level of social media use intensity. In other words, moderate social media use is associated with higher levels of social confidence, but as use intensity increases further, its positive association weakens and begins to decline (see Figure 7).

**Table 8:** Relationship between social media use intensity and the social confidence index among young adults.

Variables	Social confidence index				
	Model 1	Model 2	Model 3	Model 4	Model 5
Linear term of social media use intensity	0.782	0.343	0.203	−0.228	−0.308
	(0.509)	(0.522)	(0.524)	(0.540)	(0.544)
Quadratic term of social media use intensity	−0.755^***^	−0.682^***^	−0.647^**^	−0.696^***^	−0.698^***^
	(0.261)	(0.261)	(0.262)	(0.267)	(0.267)
Year 2023	9.159^***^	9.220^***^	8.775^***^	7.614^***^	7.301^***^
	(0.920)	(1.081)	(1.094)	(1.147)	(1.797)
Male		1.368^*^	1.368^*^	1.495^*^	1.491^*^
		(0.822)	(0.821)	(0.822)	(0.822)
Birth cohort (ref: 1970s)					
1980s		−2.212^**^	−2.146^**^	−2.247^**^	−2.188^**^
		(1.088)	(1.087)	(1.087)	(1.088)
1990s		0.094	0.111	−0.283	−0.170
		(1.412)	(1.412)	(1.415)	(1.417)
2000s		0.060	0.041	−0.635	−0.463
		(2.552)	(2.551)	(2.555)	(2.559)
Urban hukou		5.061^***^	4.476^***^	3.273^***^	3.142^***^
		(0.869)	(0.898)	(0.961)	(0.970)
Han ethnicity		3.339^**^	3.186^**^	2.972^**^	2.558^*^
		(1.373)	(1.373)	(1.376)	(1.417)
Socioeconomic status index (ISEI)			0.056^***^		0.018
			(0.022)		(0.024)
Educational attainment (ref: primary school or below)					
Junior high school				−0.404	−0.531
				(1.587)	(1.591)
Senior high school				1.964	1.616
				(1.814)	(1.837)
Associate degree or above				5.047^***^	4.278^**^
				(1.835)	(1.953)
Log of GDP per capita					1.706
					(1.308)
Log of local fiscal healthcare expenditure per capita					−0.978
					(1.839)
Constant	36.266^***^	31.229^***^	29.321^***^	30.193^***^	33.999^***^
	(0.702)	(1.764)	(1.912)	(2.201)	(11.188)
*N*	5,228	5,228	5,228	5,228	5,228

^***^, ^**^, and ^*^ denote 1%, 5%, and 10% significance levels, respectively.

With respect to gender, birth cohort, hukou, and ethnicity, the social confidence index exhibits relatively clear group differences. The coefficient on gender is significantly positive in Models 2 through 5, indicating that male young adults have significantly higher levels of social confidence than female young adults. In terms of birth cohort, with those born in the 1970s as the reference group, respondents born in the 1980s have significantly lower social confidence index scores across all models, suggesting that this cohort difference is relatively stable. By contrast, although the coefficients for those born in the 1990s and 2000s fluctuate across models, neither reaches statistical significance. The coefficients on hukou and ethnicity are both significantly positive in Models 2 through 5, indicating that young adults with urban hukou and Han ethnicity generally exhibit higher levels of social confidence. Meanwhile, the year variable is significantly positive in all models, indicating that, compared with 2013, young adults in 2023 have significantly higher social confidence index scores.

With respect to socioeconomic status and educational attainment, the coefficient on ISEI is significantly positive in Model 3 (β = 0.056, *p* < 0.01), indicating that higher socioeconomic status is associated with a higher social confidence index among young adults. However, once educational attainment and province-level macro variables are simultaneously included in Model 5, the coefficient on ISEI declines to 0.018 and is no longer statistically significant, suggesting that socioeconomic status has limited independent explanatory power for young adults' social confidence. By contrast, the association with educational attainment is more robust. Using young adults with primary school education or below as the reference group, those with junior college education or above exhibit significantly higher levels of social confidence in both Models 4 and 5. Although the coefficients for young adults with junior high school and senior high school education are positive, they do not reach statistical significance in the full model. These findings suggest that higher educational attainment—especially tertiary education—is robustly associated with stronger social confidence.

With respect to the province-level macro variables, Model 5 shows that neither the coefficient on the log of GDP per capita nor the coefficient on the log of local fiscal healthcare expenditure per capita reaches statistical significance.

Overall, social media use intensity exhibits a significant inverted U-shaped relationship with the social confidence index among young adults. Further robustness checks show that this non-linear relationship persists when the key explanatory variable is replaced by leisure-time internet use intensity. Based on the estimated turning point, the turning point for leisure-time internet use intensity is approximately 3.59 on the original 1–5 scale, with the peak occurring roughly between “several times a month” and “several times a week.” When the key explanatory variable is replaced by primary source of information, young adults who rely on the internet as their primary source of information have significantly higher social confidence index scores than those who rely primarily on other channels.

### 5.3 Heterogeneous association between internet use and young adults' mental health

Table 9 presents the regression results for the social emotion index, including social media use, the control variables, and their interaction terms. Due to space constraints, we report only interaction effects that are statistically significant. In the regression analysis, we sequentially add each control variable and its interaction with the social media use intensity. The results show that in models including only the focal independent variable, the controls, and their interaction terms, the coefficient on social media use intensity varies by survey year, educational attainment, birth cohort, and socioeconomic status (ISEI). After all variables are included simultaneously, the interaction with educational attainment is no longer statistically significant, whereas the interactions with gender and hukou status remain statistically significant.

**Table 9:** Heterogeneity in the association between social media use and the social emotion index.

Variables	Social emotion index				
	Model 1	Model 2	Model 3	Model 4	Model 5
Social media use intensity	1.79^***^ (0.170)	1.31^***^ (0.450)	2.41^***^ (0.260)	2.19^***^ (0.340)	1.61^***^ (0.620)
Male					0.09 (0.330)
Birth cohort (ref: 1970s)					
1980s		−1.50^***^ (0.440)			−1.41^***^ (0.440)
1990s		−0.22 (0.520)			−1.21^**^ (0.600)
2000s		−1.46 (0.940)			−1.51 (1.550)
Urban hukou					0.12 (0.400)
Educational attainment (ref: primary school or below)					
Junior high school			0.91 (0.820)		2.04^**^ (0.99)
Senior high school			2.23^***^ (0.820)		3.27^***^ (1.00)
Associate degree or above			4.70^***^ (0.810)		4.49^***^ (1.04)
Socioeconomic status index (ISEI)				0.07^***^ (0.01)	0.03^***^ (0.010)
Social media use intensity × Male					0.53^*^ (0.310)
Social media use intensity × Cohort					
Social media use intensity × 1980s			−0.81 (0.520)		−0.57 (0.370)
Social media use intensity × 1990s			−1.32^**^ (0.590)		−1.41^**^ (0.650)
Social media use intensity × 2000s			−0.98^**^ (0.590)		−0.63 (2.02)
Social media use intensity × Urban hukou					1.10^**^ (0.430)
Social media use intensity × Education					
Social media use intensity × Junior high school					−0.15 (0.600)
Social media use intensity × High school					−0.42 (0.710)
Social media use intensity × Associate degree or above					−0.10 (0.840)
Social media use intensity × ISEI				−0.03^***^ (0.01)	−0.03^**^ (0.010)
Year 2023	−9.93^***^ (0.360)	−11.18^***^ (0.320)	−10.36^***^ (0.370)	−8.19^***^ (0.400)	−7.50^***^ (0.530)
Social media use intensity × Year 2023	−2.07^***^ (0.440)				−1.21^*^ (0.66)
Constant	50.58^***^ (0.190)	48.04^***^ (0.770)	51.51^***^ (0.360)	47.81^***^ (0.400)	46.82^***^ (1.02)
*N*	6,356	6,356	6,356	4,440	4,440

^***^, ^**^, and ^*^ denote 1%, 5%, and 10% significance levels, respectively.

**Figure 8:**
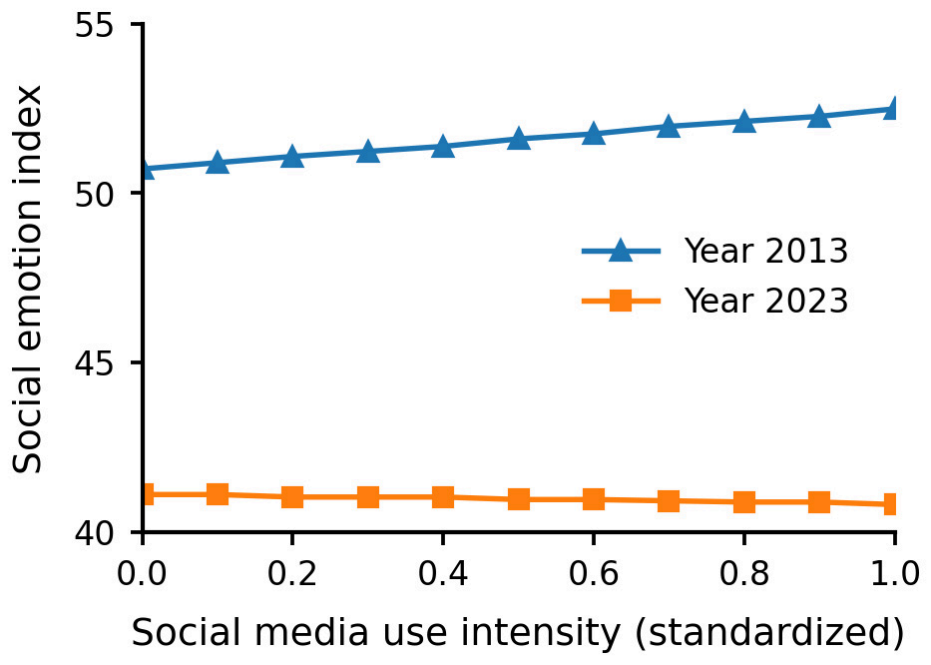
Cohort differences in the effect of social media use on the social emotion index.

Our findings indicate that, first, compared with 2013, the positive association between the social media use intensity and the social emotion index is weaker in 2023 (β = −2.07, *p* < 0.01; see Figure 8). Relative to 2013, the online environment in 2023 has undergone structural changes across multiple dimensions, imposing heavier and more complex emotional burdens on young adults. Information transmission has become more fragmented, continuously competing for attention and undermining sustained concentration. Meanwhile, highly curated portrayals and performances of everyday life by strangers on social media intensify negative social comparisons and reduce perceived gains, while emotional and one-sided content is further amplified through social media dissemination.

**Figure 9:**
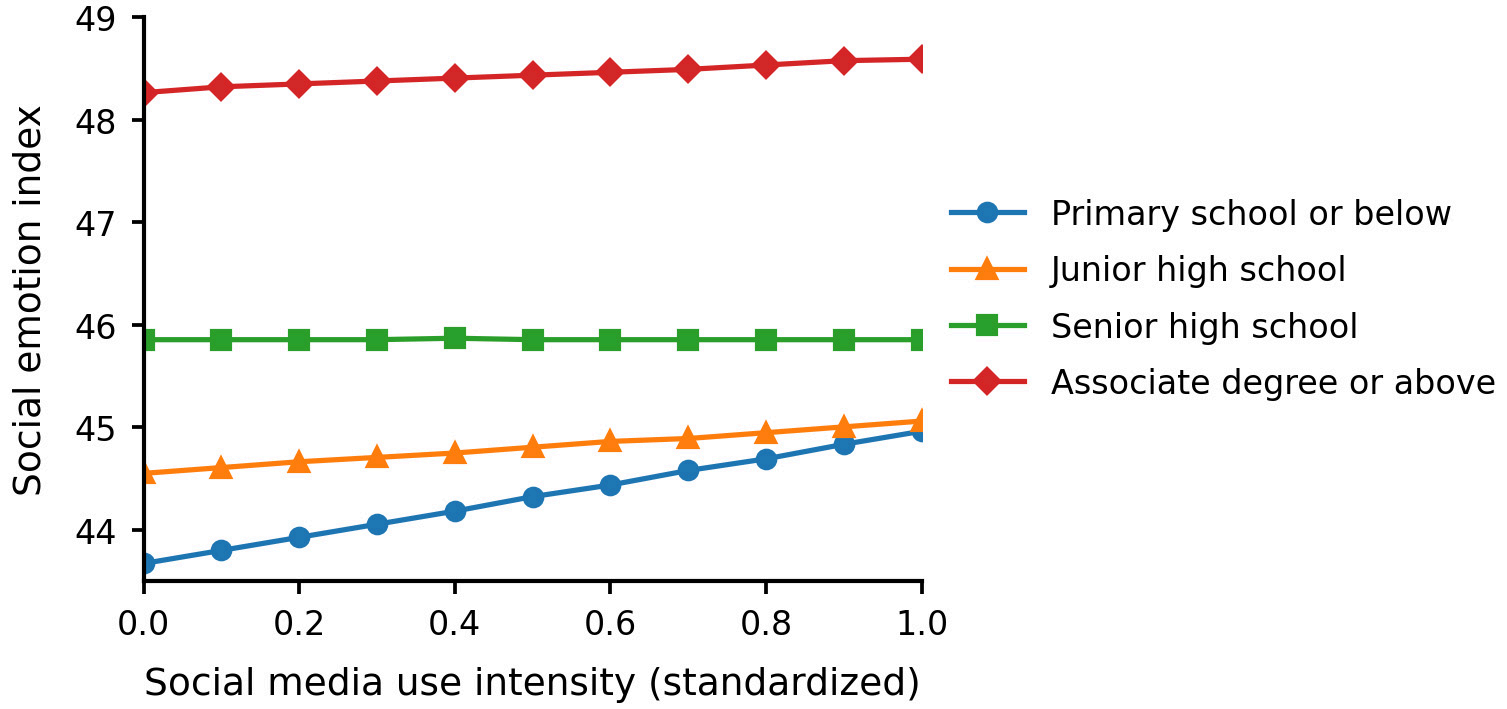
Educational differences in the effect of social media use on the social emotion index.

Second, relative to respondents with primary school education only, the positive association between social media use intensity and the social emotion index is smaller among those with a high school education and those with an associate degree or higher (social media use intensity × high school: β = −1.32, *p* < 0.05; social media use intensity × associate degree or higher: β = −0.98, *p* < 0.10; see Figure 9). Young adults with higher educational attainment typically have stronger capacities for screening and processing information; they use social media to access a broader range of information, engage with more diverse social realities, and, in doing so, develop deeper understanding and reflection on their own lives. By contrast, young adults with lower educational attainment may be more likely to use social media primarily for entertainment and basic social interaction, receive information in a more direct manner, and thus obtain stronger short-term emotional rewards from social media use.

**Figure 10:**
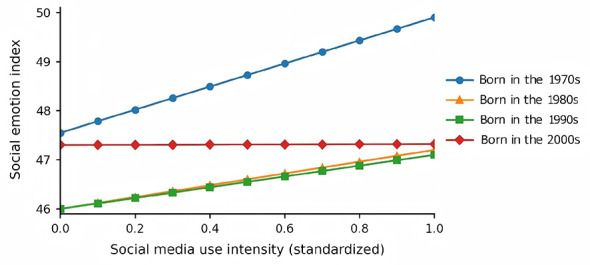
Generational differences in the effect of internet use on the social emotion index.

Third, relative to the 1970s birth cohort, the positive association between social media use intensity and the social emotion index is weaker among the 1980s and 1990s cohorts (social media use intensity × 1980s: β = −1.17, *p* < 0.01; social media use intensity × 1990s: β = −2.43, *p* < 0.01; see Figure 10). Individuals born in the 1970s gradually adopted social media during China's digital transformation and are more likely to treat it as a supplementary tool that enhances everyday life. By contrast, for young adults born in the 1980s and 1990s, social media use is deeply embedded in daily routines; during key life-course transitions, online spaces filled with class comparisons, success narratives, and uncertainty-related information may more readily elicit anxiety.

**Figure 11:**
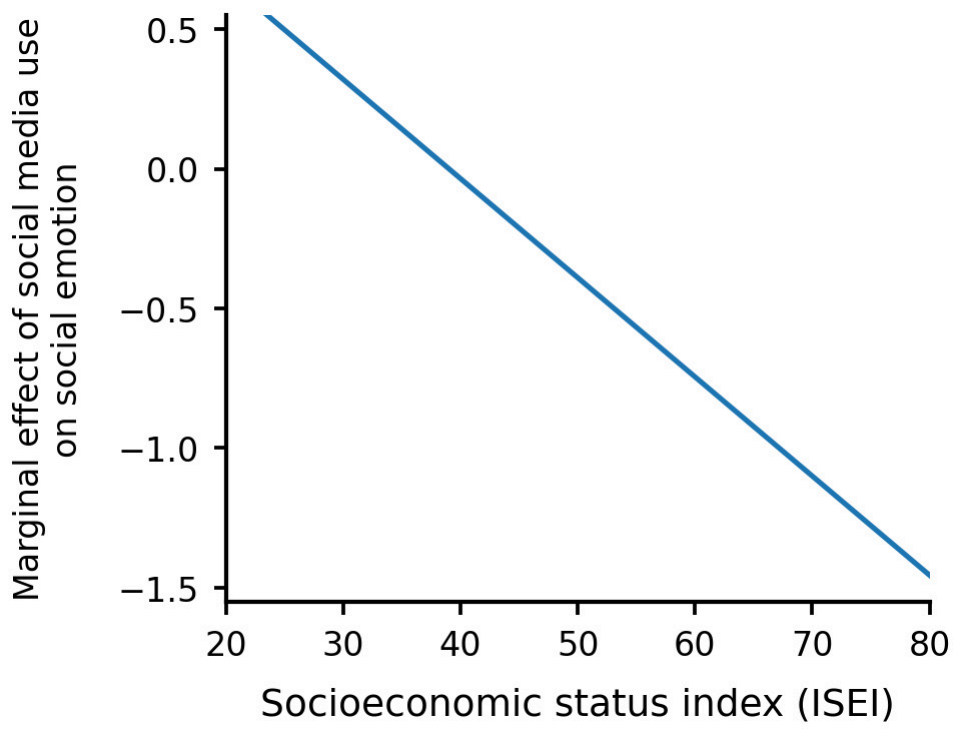
Socioeconomic-status differences in the effect of internet use on the social emotion index.

Fourth, higher socioeconomic status weakens the positive association between social media use and the social emotion index (β = −0.03, *p* < 0.01; see Figure 11). Young adults with higher socioeconomic status have greater economic resources. Amid the rapid expansion of China's cultural and entertainment service industries, they often enjoy richer and more diverse offline experiences. Greater satisfaction with offline life means that these young adults are less likely to rely on social media use for emotional fulfillment; instead, they tend to view social media primarily as a tool for information acquisition or improving efficiency, resulting in smaller emotional gains from social media use.

**Figure 12:**
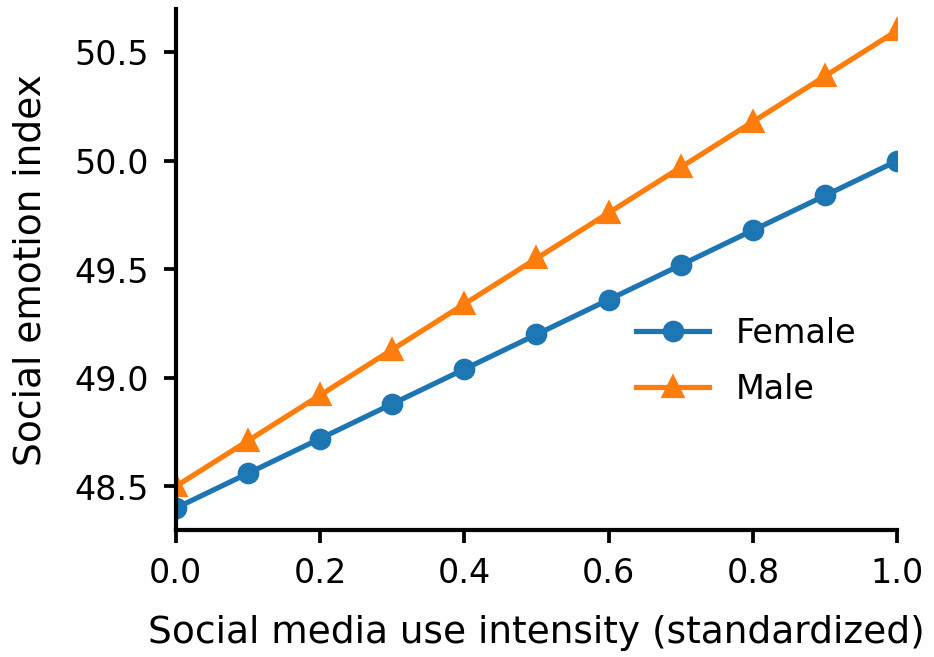
Gender differences in the effect of internet use on the social emotion index.

Fifth, compared with young women, young men show a stronger positive association between social media use intensity and the social emotion index (β = 0.53, *p* < 0.10; see Figure 12). Several mechanisms may account for this pattern. First, young men and young women may be exposed to different types of information and social environments on social media, leading to different emotional experiences. Second, they may differ in their primary purposes for using social media: young men may be more likely to use it for instrumental purposes such as information seeking and entertainment, whereas young women may be more inclined to use it to maintain social ties and seek emotional support. Finally, gender-related issues in offline settings can be reproduced and amplified online.

**Figure 13:**
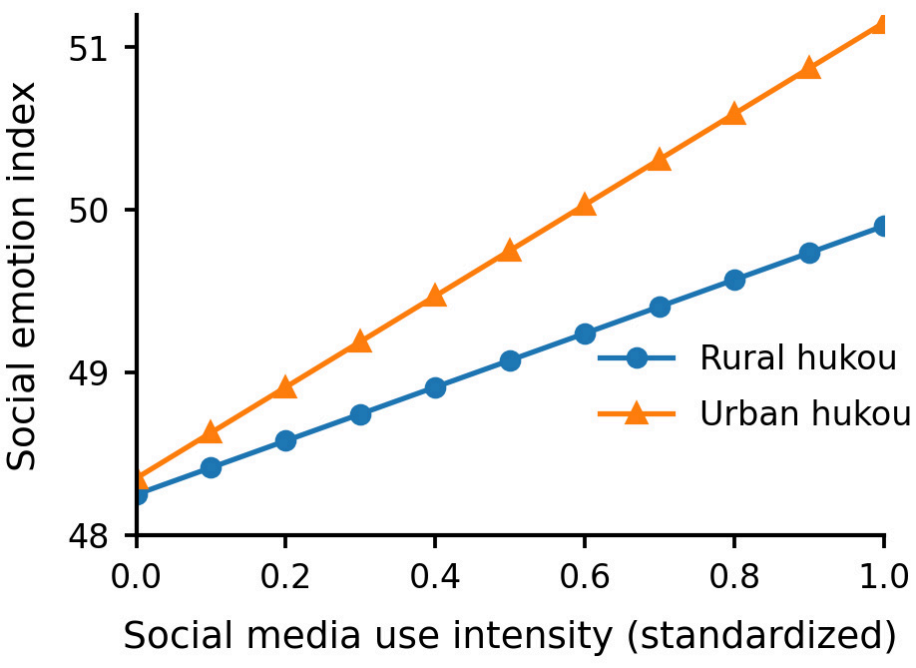
Hukou-based differences in the effect of internet use on the social emotion index.

Sixth, compared with young adults with rural hukou, those with urban hukou exhibit a stronger positive association between social media use intensity and the social emotion index (β = 1.10, *p* < 0.05; see Figure 13). Urban young adults may be exposed to social media earlier, have stronger capacities to access, evaluate, and integrate information, and be more likely to express their views online. In turn, they may contribute to shaping online spaces that better reflect urban youth's preferences and norms. Cultural differences between urban and rural youth may further underlie hukou-based heterogeneity in the association between social media use and the social emotion index.

When we replaced the focal independent variable with the social media use intensity during leisure time or with primary source of information (internet = 1), the conclusions remained unchanged.

## 6 Conclusion

Digital transformation is one of the most pervasive social changes of the 21st century on a global scale. With the rapid development of internet and information technologies, profound changes have taken place in China's economic activities, including production, distribution, and consumption, as well as in social practices such as public governance and cultural communication. As digital natives, young adults have integrated internet use into every aspect of daily life, and young adults' mental health has also been profoundly influenced by internet use. Against this backdrop, drawing on data from the 2013 and 2023 waves of the Chinese General Social Survey (CGSS) and official statistics from the National Bureau of Statistics of China, this study focuses on young adults aged 18–40. The study constructs a social emotion index based on three dimensions—life satisfaction, social trust, and social confidence—and examines the impact of digital transformation in China, especially internet use, on young adults' mental health. The main findings are threefold.

### 6.1 An overall decline in young adults' mental health from 2013 to 2023

Compared with 2013, the social emotion index among young adults in China declined significantly in 2023. In particular, the life satisfaction component dropped markedly, driven mainly by a pronounced increase in negative emotions and work-related stress among young adults. Although young adults' subjective wellbeing has improved with better material living conditions and a more comprehensive social security system, the principal contradiction facing Chinese society is the contradiction between unbalanced and inadequate development and the people's ever-growing needs for a better life. Accordingly, young adults' life goals have shifted from meeting basic needs—such as forming a family and securing stable employment—to pursuing higher-order aspirations centered on autonomy, self-fulfillment, and diverse life possibilities. Within China's digital transformation, young adults are exposed to a wider range of perspectives online. These diverse narratives—especially portrayals of others' “better lives”—can heighten young adults' aspirations and strengthen their motivation for upward mobility. At the same time, however, digital transformation has brought profound socioeconomic restructuring: traditional employment pathways are being disrupted, emerging labor markets have not yet fully consolidated, and pressures on young adults' livelihoods and life-course development have intensified. Meanwhile, both the social trust index and the social confidence index increased significantly. This pattern suggests that young adults express strong confidence in governance effectiveness, the stability of social order, and ongoing efforts to advance social fairness, and it is consistent with a generally optimistic outlook on future development. In contemporary China, young adults' pursuit of a better life increasingly unfolds alongside mounting work pressure and anxiety.

### 6.2 A significant and robust non-linear relationship between internet use and young adults' mental health

The findings show that the association between internet use and young adults' mental health is neither a simple linear enhancement nor a simple linear inhibition; rather, it follows a significant and robust inverted U-shaped pattern. Whether measured by the overall social emotion index or by the two subdimensions of life satisfaction and social confidence, moderate or moderately high levels of internet use are associated with more positive mental health outcomes. However, once the intensity of internet use increases further, its positive association weakens markedly and begins to decline. Young adults who rely on the internet as their primary source of information also exhibit more positive mental health than those who rely primarily on other channels for information. These findings suggest that moderate internet use can help young adults expand social connections, access information resources, obtain emotional support, and, to some extent, relieve pressures in everyday life. Once internet use becomes excessive, however, it may crowd out time for offline interaction and rest, reinforce social comparison and emotional contagion, and thereby adversely affect young adults' mental health. It should also be noted that internet use does not exhibit a significant and robust relationship with young adults' social trust. This suggests that the association between internet use and young adults' mental health are more evident in dimensions related to lived experience, subjective evaluation, and expectations for the future, while its influence on social trust, which is more structural and cognitive in nature, remains relatively limited.

### 6.3 The association between internet use and young adults' mental health varies across subgroups

Our analyses further reveal substantial heterogeneity in the association between internet use and young adults' mental health. Across survey years, the positive association between internet use and young adults' mental health is weaker in 2023 than in 2013. In terms of gender, the positive association between internet use and young adults' mental health is larger for men than for women. One plausible interpretation is that women are more likely to use the internet to sustain social ties and seek emotional support, whereas men more often use the internet for instrumental purposes such as information seeking and entertainment, which may yield more immediate emotional benefits. Heterogeneity is also evident by hukou status: compared with young adults holding rural hukou, those with urban hukou experience a stronger positive association between internet use and young adults' mental health. Urban young adults may have been exposed to social media earlier and, through online participation and expression, have helped shape an online environment more aligned with urban youth culture. By contrast, among young adults with at least a high school education, higher socioeconomic status, and younger cohorts, the positive association between internet use and young adults' mental health is attenuated. These patterns suggest that for young adults who are more deeply embedded in digital life, face more intense competition in daily life, and possess greater capacity to evaluate and filter information, internet use has limited potential to further enhance mental health and, in turn, young adults' mental health.

Overall, digital transformation has accelerated the pace of contemporary young adults' lives. Constant connectivity has blurred the boundary between public and private life. Intensified social competition and information overload have become increasingly salient. At the same time, socioeconomic restructuring has also contributed to greater employment instability among young adults. These structural changes have broadly heightened young adults' anxiety, stress, and sense of uncertainty, contributing to an overall decline in mental health. However, the expansion of the internet—the core engine of digital transformation—also provides young adults with crucial adaptive space. Through internet use, young adults can access broader forms of social support, seek emotional resonance, and relieve negative emotions, making the internet an important platform and arena for coping with real-world pressures. In the context of digital transformation, internet use has become an important “safe haven” for young adults' mental health.

## Data Availability

Publicly available datasets were analyzed in this study. This data can be found here: https://www.cnsda.org/index.php?r=projects/index.

## Footnotes

1 This study focuses on young adults aged 18–40. In the analysis based on the 2013 data, three birth cohorts are distinguished: those born in the 1970s, 1980s, and 1990s; in the analysis based on the 2023 data, three cohorts are defined as those born in the 1980s, 1990s, and 2000s. In the statistical models, the oldest cohort (those born in the 1970s and 1980s, respectively) is used as the reference group.

2 Source: China Internet Network Information Center (CNNIC).

3 During the PCA, work pressure and subjective expectations of social mobility were initially included. Given their relatively low loadings on the principal components and their limited ability to reflect the underlying dimensions, both variables were excluded from the final model.
